# Identifying Game-Based Digital Biomarkers of Cognitive Risk for Adolescent Substance Misuse: Protocol for a Proof-of-Concept Study

**DOI:** 10.2196/46990

**Published:** 2023-11-23

**Authors:** Kammarauche Aneni, Ching-Hua Chen, Jenny Meyer, Youngsun T Cho, Zachary Chase Lipton, Saatvik Kher, Megan G Jiao, Isabella Gomati de la Vega, Feza Anaise Umutoni, Robert A McDougal, Lynn E Fiellin

**Affiliations:** 1 Child Study Center Yale School of Medicine New Haven, CT United States; 2 Biomedical Informatics and Data Science Yale School of Medicine New Haven, CT United States; 3 Center for Computational Health IBM Research Yorktown Heights, NY United States; 4 Fairfield University Fairfield, CT United States; 5 Department of Psychiatry Yale School of Medicine New Haven, CT United States; 6 Machine Learning Department School of Computer Science Carnegie Mellon University Pittsburg, PA United States; 7 Pomona College Claremont, CA United States; 8 McGovern Medical School UTHealth Houston Houston, TX United States; 9 Pontificia Universidad Javeriana Bogota Colombia; 10 Yale School of Public Health New Haven, CT United States; 11 Department of Internal Medicine Yale School of Medicine New Haven, CT United States

**Keywords:** game-based digital biomarkers, cognition, adolescent substance misuse, cognitive risk factors, game-based measurement of cognitive function, biomarker, adolescent, screening, game, barrier, digital, substance, use, misuse

## Abstract

**Background:**

Adolescents at risk for substance misuse are rarely identified early due to existing barriers to screening that include the lack of time and privacy in clinic settings. Games can be used for screening and thus mitigate these barriers. Performance in a game is influenced by cognitive processes such as working memory and inhibitory control. Deficits in these cognitive processes can increase the risk of substance use. Further, substance misuse affects these cognitive processes and may influence game performance, captured by in-game metrics such as reaction time or time for task completion. Digital biomarkers are measures generated from digital tools that explain underlying health processes and can be used to predict, identify, and monitor health outcomes. As such, in-game performance metrics may represent digital biomarkers of cognitive processes that can offer an objective method for assessing underlying risk for substance misuse.

**Objective:**

This is a protocol for a proof-of-concept study to investigate the utility of in-game performance metrics as digital biomarkers of cognitive processes implicated in the development of substance misuse.

**Methods:**

This study has 2 aims. In aim 1, using previously collected data from 166 adolescents aged 11-14 years, we extracted in-game performance metrics from a video game and are using machine learning methods to determine whether these metrics predict substance misuse. The extraction of in-game performance metrics was guided by literature review of in-game performance metrics and gameplay guidebooks provided by the game developers. In aim 2, using data from a new sample of 30 adolescents playing the same video game, we will test if metrics identified in aim 1 correlate with cognitive processes. Our hypothesis is that in-game performance metrics that are predictive of substance misuse in aim 1 will correlate with poor cognitive function in our second sample.

**Results:**

This study was funded by National Institute on Drug Abuse through the Center for Technology and Behavioral Health Pilot Core in May 2022. To date, we have extracted 285 in-game performance metrics. We obtained institutional review board approval on October 11, 2022. Data collection for aim 2 is ongoing and projected to end in February 2024. Currently, we have enrolled 12 participants. Data analysis for aim 2 will begin once data collection is completed. The results from both aims will be reported in a subsequent publication, expected to be published in late 2024.

**Conclusions:**

Screening adolescents for substance use is not consistently done due to barriers that include the lack of time. Using games that provide an objective measure to identify adolescents at risk for substance misuse can increase screening rates, early identification, and intervention. The results will inform the utility of in-game performance metrics as digital biomarkers for identifying adolescents at high risk for substance misuse.

**International Registered Report Identifier (IRRID):**

DERR1-10.2196/46990

## Introduction

### Background

Despite existing evidence-based interventions, many adolescents continue to misuse substances [1]. In 2021 alone, over 1 in 5 adolescents aged 12-17 years misused substances (illicit drugs and alcohol or tobacco products) [2] and 2 million adolescents had a substance use disorder [2]. Adolescent substance misuse, defined as “the unhealthy use of alcohol or drugs to relieve stress, alter reality or bring about pleasure” [3], increases the risk of developing a substance use disorder. Adolescents with substance use disorders are more likely to engage in criminal activity, contract sexually transmitted infections, develop comorbid mental disorders, and die early [4]. In addition, between 2019 and 2020, there was a 44% increase in drug-involved deaths among adolescents aged 10-19 years [5]. The negative consequences arising from substance misuse underscore the need to identify at-risk adolescents early to deliver timely prevention and treatment interventions.

Less than 10% of adolescents in need of substance use treatment receive it [6] in the setting of poor screening uptake and referral for treatment [7]. Many adolescents with substance misuse are not identified early, and barriers in screening adolescents for substance misuse contribute to this. The American Academy of Pediatrics and the Substance Abuse and Mental Health Services Administration recommend universal screening to identify at-risk adolescents. Yet, fewer than half of primary care providers [8] and fewer than a fifth of School-Based Health Center providers [9] screen adolescents for substance misuse [8,9]. Identifying at-risk adolescents early and delivering effective prevention interventions can prevent substance misuse-associated morbidity and mortality [10,11]. Barriers to screening adolescents for substance misuse include the lack of time, concern that adolescents will not be forthcoming about misuse, discomfort with conducting screening, and difficulty with providing a confidential space to conduct screening [8,12-15]. Innovative solutions that address or mitigate these barriers and lead to early identification of at-risk adolescents are urgently needed.

Digital tools hold great potential to mitigate identified barriers in screening for substance misuse given the ubiquity of mobile devices. These digital tools can be accessed anywhere and include mobile phones, wearables such as smart watches or Fitbits, mobile or web-based apps, and game software. Serious games—“games designed for a primary purpose other than play” [16]—are frequently used by over 95% of adolescents [17]. Serious games are increasingly being used for delivering health interventions among adolescents [18,19] and, more recently, are also being used for assessment of mental health outcomes [20,21]. During gameplay, in-game metrics reflective of performance in the game are being collected by the game software [18,22]. These metrics may reflect digital biomarkers of health outcomes [22].

Digital biomarkers are “consumer-generated physiological and behavioral measures collected through connected digital tools that explain, influence or predict health-related outcomes” [22,23]. Although most studies investigating the utility of digital biomarkers for health screening and monitoring have focused more on data collected from mobile phones [24,25] and wearables [26,27], games offer another source of data that could potentially measure digital biomarkers [22]. In-game performance metrics [18], such as the amount of time to complete a game task, accuracy of choices in a game task, or style of gameplay (ie, choosing a game task without attention to outlined rules), may reflect cognitive processes such as working memory, processing speed, and inhibitory control [22]. These cognitive processes (working memory and inhibitory control) affect in-game performance and can therefore be measured using in-game performance metrics [28,29]. Deficits in working memory [30,31] and inhibitory control [32] are also associated with substance misuse among adolescents [33] and may explain by up to 33% of the variance in future risk of substance misuse [33]. As such, in-game performance metrics may reflect digital biomarkers of cognitive function, which may be used in predicting adolescents at risk for substance misuse. Digital biomarkers have been used to predict health risk among adults [34,35] and adolescents [25,27]. Prior studies have also investigated the utility of digital biomarkers for a range of psychiatric disorders [24,26,34,36,37]. In these studies, the digital biomarkers captured were primarily from phone or wearable sensors and included measures such as movement, location, sleep, heart rate, and social interaction through call or text logs [24-27,34,37]. Although games are increasingly being used for measuring health outcomes such as cognitive function [38], their utility as digital biomarkers is yet to be widely investigated. In addition, all of the studies that have investigated the utility of game-based digital biomarkers have focused on adults [21,39,40]. A study by Dechant et al [39] demonstrated that participants with social anxiety exhibited in-game behavior that was different compared to participants without social anxiety. Specifically, participants with social anxiety were more likely to have their avatar stand at a larger distance to nonplayer characters in the game. In another study by Gielis et al [40], they investigated the utility of in-game performance metrics to identify adults with mild cognitive impairment (MCI). Adults identified as having MCI took more time to think about a move and made more errors in the game compared to adults without MCI.

To the best of our knowledge, we are not aware of any studies that have investigated the utility of game-based digital biomarkers for adolescent substance misuse. Given the easy access to games and the high level of motivation and engagement with games among adolescents [17,41,42], game-based digital biomarkers hold promise for risk identification among adolescents. The use of games as a screening tool could also mitigate barriers in screening since these games can be deployed without supervision and can be completed in private. In addition, prior studies have indicated that adolescents are more forthcoming when completing digital screeners compared to provider interviews and prefer this form of screening [43]. Finally, future apps of game-based screening and monitoring tools could integrate with the electronic health record to make results readily accessible to providers.

### Study Objectives

This paper describes the protocol for a proof-of-concept study to develop and test a predictive model for identifying adolescents at risk for substance misuse using in-game performance metrics. The primary objective of this study is to identify game-based digital biomarkers of cognitive risk factors implicated in the development of substance misuse among adolescents using available in-game performance metrics from an existing serious video game intervention, *PlayForward: Elm City Stories*, developed by the play2PREVENT Laboratory in partnership with Schell Games (hereafter referred to as *PlayForward*) [19].

## Methods

### Overall Study Design

The purpose of this pilot study is to identify game-based digital biomarkers of cognitive risk factors implicated in adolescent substance misuse by developing and testing a machine learning predictive model. This study is led by a team with expertise in machine learning, game design, cognitive function, adolescent substance misuse, and the conduct of pilot trials. This study’s team demonstrates a team science approach to solving public health problems with investigators from Yale University, Carnegie Mellon University, and International Business Machines Corporation.

### Aim 1

#### Objective

The objective of aim 1 is to identify game-based digital biomarkers of adolescent substance misuse.

#### Overall Study Design of Aim 1

Using data collected in a previous study [19], we will determine specific in-game performance metrics—digital biomarkers—that more strongly associate with a high risk for substance misuse. Adolescents will be grouped in 2 groups—high and low risk for substance misuse—based on their baseline responses to questions about substance misuse behaviors (eg, “in the past 30 days, have you used cannabis?” and “How sure are you that you can refuse if a friend offers you alcohol and you do not want it?”). We will have 2 prediction tasks (substance misuse and self-efficacy to refuse drugs). For each task, we will identify an effective prediction model by testing 6 machine learning classification models (eg, logistic regression, random forest, decision trees, support vector machine, gradient boosted decision tree, and neural networks). We will assess the feasibility of using a logistic regression model to compute a digital biomarker score for each adolescent that ranges from 0 to 1, where an adolescent with a higher score has a higher substance misuse risk.

#### Data Source

*PlayForward* is an evidence-based video game intervention developed in the play2PREVENT laboratory for HIV prevention and reduction of high-risk behaviors such as substance misuse [18,19,44,45]. *PlayForward* includes 12 Challenge Stacks or levels (the narrative and story-based components of the game) and 5 minigames (People Sense, Refusal Power, Me Power, Priority Sense, and Know Sense). Each of the minigames has 10 levels. The minigames are designed to help the adolescent develop and practice adaptive skills—such as learning to say no to an invitation to use drugs—that can be translated into real life. Players earn stars and points during gameplay, which allow them to advance in the game. *PlayForward* was tested in a randomized controlled trial of 333 adolescents, aged 11-14 years, where 166 adolescents were assigned to play the *PlayForward* video game [19]. At baseline, self-report data on substance misuse and self-efficacy to refuse drugs were collected. During gameplay, data related to their performance in the game (Table 1) were collected by the game software and later stored as log files. The game developers (Schell Games) developed reference files for decoding information stored in the log files.

**Table 1:** Potential in-game biomarkers.

Minigame or subsection of minigame		Potential in-game biomarkers		Hypothesized neurocognitive domains (DSM-5^a^)
**People sense**				
	Deciding friends: checking out each person’s information	Time spent checking peer’s characteristics Correctly sorting peers into the right social or friendship circles Time spent completing social or friendship circles	Working memory, attention, inhibitory control, and processing speed Working memory and decision-making Working memory and processing speed	
	Accepting or rejecting invites	Correctly accepting or declining invitations based on risk and social circles Proportion of accepted unsafe invitations	Working memory, decision-making, and judgement Decision-making, and judgement	
**Refusal power**				
	Think	Correctly identifying the type of peer pressure	Decision-making and inhibitory control	
	Prepare	Correctly choosing reasons to say no	Decision-making and inhibitory control	
	Refuse	Time spent completing the sentence portion of minigame	Working memory and processing speed	
Priority sense		Number of times priority dropped to zero		Working memory
Know sense		Total credibility score at the end of the minigame		Working memory, decision-making, and processing speed

^a^DSM-5: Diagnostic and Statistical Manual, 5th Edition.

#### Data Extraction and Analysis

Three authors (KA, MGJ, and IGdlV) played the *PlayForward* game and reviewed the literature and gameplay guides for *PlayForward* to identify in-game performance metrics that likely used cognitive function and could therefore be potential in-game digital biomarkers of cognitive function (Table 1). We have identified 285 in-game performance metrics (eg, the number of times the player lost the minigame; Multimedia Appendix 1). One of the authors (SK) extracted all identified in-game performance metrics from log files into a CSV file and uploaded this into R statistical software (R Foundation for Statistical Computing) [46]. In-game performance metrics that are found to predict substance misuse risk following analysis will be identified as digital biomarkers for adolescent substance misuse risk.

In total, 2 outcomes (described in the assessments section below)—substance misuse and self-efficacy—to refuse drugs will be used in 2 prediction models. Based on adolescent baseline response to questions on substance misuse and self-efficacy to refuse drugs in a previous study [19], a substance misuse score and self-efficacy to refuse drugs score have been derived. For each score, adolescent participants are categorized into 2 groups (high risk and low risk) based on the score distribution. A preliminary examination of the log files of the 166 participants who played the *PlayForward* game resulted in dropping 6 log files due to file errors. As such, analysis of aim 1 will occur with a final sample of 160 participants.

#### Outcome Measures

##### Substance Misuse

In total, 20 questions on cigarette, alcohol, and drug use behaviors from the Youth Risk Behavior survey [47] were administered to 11- to 14-year-old adolescents in the *PlayForward* randomized controlled trial at baseline [19].

Questions included whether participants had ever tried using cigarette, alcohol, or other drugs (marijuana, cocaine; inhalants; over-the-counter drugs; and nonprescribed prescription drugs such as opioids, methamphetamines, and injection drugs); substance use in the previous 30 days; and age at first use (for cigarette and alcohol).

In total, 15 questions on ever use and past 30-day use (for substances other than alcohol or cigarette) had options of yes, no, and decline to answer. In total, 2 questions on age at first use (for cigarette and alcohol) had 9 options: “I have never smoked a whole cigarette/I have never had a drink of alcohol other than a few sips”; 8, 9, 10, 11, 12, 13, and 14 years or older; and decline to answer. Further, 1 question on past 30-day use of cigarettes had 8 options: I did not smoke cigarettes during the past 30 days, less than 1 cigarette/day, 1 cigarette per day, 2-5 cigarettes/day, 6-10 cigarettes/day, 11-20 cigarettes/day, more than 20 cigarettes/day, and decline to answer. Furthermore, 1 question on past 30-day use of alcohol had 8 options: 0 days, 1 day, 2 days, 3-5 days, 6-9 days, 10-19 days, 20 or more days, and decline to answer.

In addition, if participants answered “no” to a particular question about having ever used cigarette or alcohol or drugs, they skipped subsequent questions about past 30-day use and age at first use (for cigarettes and alcohol). A participant could skip up to 9 questions.

##### Coding

For this age group, we considered any type of substance misuse to be high risk. As such, if any adolescent answered “yes” to any of the above questions, we coded them as high risk. Based on this criterion, 107 (66.9%) of 160 adolescents were low risk because they answered “no” to all the substance misuse questions.

##### Self-Efficacy to Refuse Drugs

In total, 10 questions from the Drug Use Resistance Self-Efficacy [48] scale were used to calculate a score. The Drug Use Resistance Self-Efficacy scale asks questions relating to confidence in one’s ability to refuse drugs in a variety of situations. The options to each question include “not sure at all,” “not very sure,” “pretty sure,” and “definitely sure.” Each option was recoded as follows: “definitely sure”=0, “pretty sure”=1, “not very sure”=2, and “not sure at all”=3. As such, scores could range from 0 to 30, and higher scores meant lower self-efficacy to refuse drugs. In this sample of 160 adolescents, scores ranged from 0 to 27 with a mean of 2.38 (SD 4.40). Adolescents were grouped into 2 groups (high risk and low risk) based on the score distribution. Adolescents with a total score of 0 were considered low risk (n=82, 51.2%), whereas adolescents with a total score greater than 0 were considered high risk (n=78, 48.8%).

#### Analyses

Machine learning models [49] will be used to predict each outcome. We will use k-fold cross-validation [50] to evaluate the performance of 6 machine learning classification models (logistic regression, random forest, decision trees, support vector machine, gradient boosted decision trees, and neural networks) to predict each of the outcomes. These 6 models are representative of a range of popular classification models that have been previously used in substance use research [51]. These models vary in their underlying mathematical approaches and often offer reasonable to good performance across a variety of data sets. For example, while regression models are easy to implement, the underlying assumption is that predictors are independent [51]. Decision trees on the other hand do not require elaborate preprocessing but perform relatively worse than other models [51]. Support vector machine performs well using unstructured data but requires extensive tuning [51]. The primary evaluation metric used will be the area under the receiver operating characteristic (AUROC) curve [50]. For each outcome, the model with the AUROC value closest to 1 is the one that has the best ability to discriminate between risk classes, across all classification threshold levels ranging from 0 to 1 [50]. Ideally, an AUROC value of greater than 0.7 can be achieved by the best model. We will also report the sensitivity and specificity for a range of classification thresholds. In the event that AUROC values are similar across candidate models, it may be useful to identify the best model as the model that offers the most appropriate trade-off between sensitivity and specificity, even if the AUROC value of that model may not be the highest [50,52]. Existing screening tools for substance use have sensitivity ranging from 0.71 to 0.94 and specificity ranging from 0.87 to 0.97 [53,54]. As such, to identify an adolescent at risk for substance misuse, we want to achieve comparable sensitivity and specificity above 0.7. Given the risk of negative outcomes such as an overdose that can occur with adolescent substance misuse, a model that produces a higher sensitivity compared to its specificity would be consistent with existing recommendations for substance use screening tools [53]. For the best model identified for each outcome, we use Shapley additive explanations [55,56] to explain the relative importance of the features used to predict the outcome class (ie, high risk vs low risk). We will define features (ie, predictor variables used to inform the classification) from in-game performance metrics. When selecting features to be used for classification, the variance threshold method will be used to remove features with 0 (or low) variance because they are unlikely to be useful for discriminating between classes [49]. We will reduce multicollinearity between features by selectively removing a subset of highly correlated features before performing classification [49]. In addition, we will assess in-game metrics for multicollinearity and drop in-game performance metrics with a variable inflation factor larger than 10 [57].

### Aim 2

#### Objective

The objective of aim 2 is to determine the association between identified digital biomarkers and cognitive function.

#### Overall Study Design of Aim 2

We will test the hypothesis that digital biomarker scores will negatively correlate with cognitive function scores. To test this hypothesis, we will conduct a pilot study using a new sample of 30 high school adolescents aged 14-15 years. After screening, consent, and baseline assessments, adolescents will play the *PlayForward* game. To validate the model developed in aim 1, the digital biomarkers identified in aim 1 will be extracted following completion of gameplay, and a digital biomarker score will be derived. We will then test the association between the computed digital biomarker score and cognitive function.

#### Participants and Source

Adolescents from 1 to 3 Connecticut high schools will be recruited. To be eligible for this study, participants must (1) attend a high school in Connecticut, (2) be aged between 14 and 15 years, (3) be willing to sit for 60 minutes per session to play the game, and (4) be able to provide assent and parental or guardian consent. Following screening, eligible adolescents who provide parental consent and assent will complete baseline assessments. Adolescents who complete baseline will be grouped into high-risk and low-risk groups based on their response to assessment of substance misuse, depression, and anxiety. High risk will be determined using their report at baseline of monthly use of any substance (alcohol, tobacco, marijuana, prescription drugs, inhalants, illegal drugs, and synthetic drugs), a score of >2 on the Patient Health Questionnaire-2 [58], and a score of >2 on the Generalized Anxiety Disorder 2-item scale [59]; low risk will be determined by no substance use in the past year, <2 on the Patient Health Questionnaire-2, and <2 on the Generalized Anxiety Disorder 2-item scale. We chose a higher age range (14-15 y) for aim 2 (in contrast to the age range of 11-14 y in aim 1) to ensure that we can recruit a high-risk sample (adolescents with substance misuse).

#### Recruitment and Informed Consent

This study will take place in high schools where the laboratory has established partnerships and has conducted trials in the past. Adolescents in participating high schools will be informed about this study. Adolescents who express interest will be provided a flyer with a QR code to complete a contact form if interested in participating in the study. Parents of interested adolescents who complete the contact form will be contacted to obtain parental permission. Following parental permission, adolescents will be screened for eligibility for participation in this study through a Qualtrics (Qualtrics) survey. Eligible adolescents will be asked to provide assent prior to enrollment into this study.

#### Data Collection and Storage

We will use Qualtrics, a web-based data collection software to obtain parental permission and adolescent assent, screen adolescents for study eligibility, obtain demographic information, and generate a study ID. Study IDs will be automatically generated after the adolescent has screened as eligible. PsyToolkit [60,61], an open source, web-based psychological instrument will be used to administer baseline assessments and conduct the tasks of cognitive function. PsyToolkit has been used in studies measuring working memory and response inhibition in adolescents [62,63] and has been shown to produce comparable results to tasks conducted in a laboratory [64]. Baseline assessment and task performance scores will be retrieved from the PsyToolkit website.

Gameplay data collection will be stored on iPads (Apple Inc) during gameplay. Following study completion, data for each participant will be retrieved from Qualtrics and PsyToolkit into a CSV file and merged by participant ID. Data from iPads will also be retrieved as log files. All retrieved data will be stored on secured data storage locations on the Yale server. Data collection will occur between April 2023 and February 2024.

#### Assessments and Cognitive Tasks

Table 2 [48,58,65-70] lists measures to be used for screening and administered at baseline. All listed measures have been validated among adolescents.

Table 3 [71-74] lists cognitive tasks to be completed by adolescents at baseline. All listed cognitive tasks have been validated among adolescents.

**Table 2:** List of screening and baseline assessments for aim 2.

Construct	Name of questionnaire and reference	Number of items	Screening	Baseline
Demographics	Age, sex, and school name	—^a^	✓	—
Depression	Patient Health Questionnaire-2, Richardson et al [58]	2	✓	—
Anxiety	Generalized Anxiety Disorder-7, Bentley et al [65]	2	✓	—
Substance misuse	Screening to Brief Intervention scale, Levy et al [66]	7	—	✓
Substance use risk	Car, Relax, Alone, Forget, Friends, Trouble (CRAFFT) scale, Knight et al [70]	6	✓	—
Emotion regulation	Difficulties in Emotion Regulation Scale–Short Form, Kaufman et al [67]	18	—	✓
Cognitive function	Teenage Executive Function Inventory Scale, Thorell et al [68]	20	—	✓
Impulsivity	Urgency, Premeditation (lack of), Perseverance (lack of), Sensation Seeking, Positive Urgency, Impulsive Behavior scale, Pechorro et al [69]	20	—	✓
Self-efficacy to refuse drugs	Drug Use Resistance Self-Efficacy scale, Carpenter and Howard [48]	24	—	✓

^a^Not applicable.

**Table 3:** Baseline cognitive tasks.

Construct or cognitive task and reference		Description	Metrics recorded
**Working memory**			
	n-back task, Pelegrina et al [71]	The 2-back task will be used. In the 2-back task, a series of letters will be shown to participants in a sequential order. Participants are to click a prespecified key on their device if the letter shown matches the letter shown 2 turns back.	Percentage of correct matches Percentage of missed matches Percentage of false alarms Mean response time
**Inhibitory control**			
	Go or no-go task, Bezdjian et al [72]	In the go or no-go task, participants are asked to click the spacebar when they see the “go” sign but do nothing when they see the “no-go” sign.	Response speed Number of errors Mean response time
	Stop-signal task, Kulendran et al [73]	In the stop signal task, 2 arrows, 1 pointing to the right and the other pointing to the left, will be shown in random order. In the first round, without the stop signal, the participant will click a prespecified key when the left pointing arrow is shown and a different prespecified key when the right pointing arrow is shown. In the second round which includes the stop signal, participants are to stop from clicking the arrows when the red signal appears.	Mean response time Error rate
**Selective attention, processing speed, and cognitive flexibility**			
	Stroop task, Stroop [74]	In the Stroop task, names of different colors (blue, green, yellow, and red) will be printed in either of the 4 colors. Participants are to use prespecified keyboard keys to identity the color in which the word is printed in and not the meaning of the word. If the word “yellow” is printed in green, the correct response would be to click on the key that corresponds to green.	Mean response time for congruent and incongruent stimuli

#### Study Setting and Study Flow

**Figure 1:**
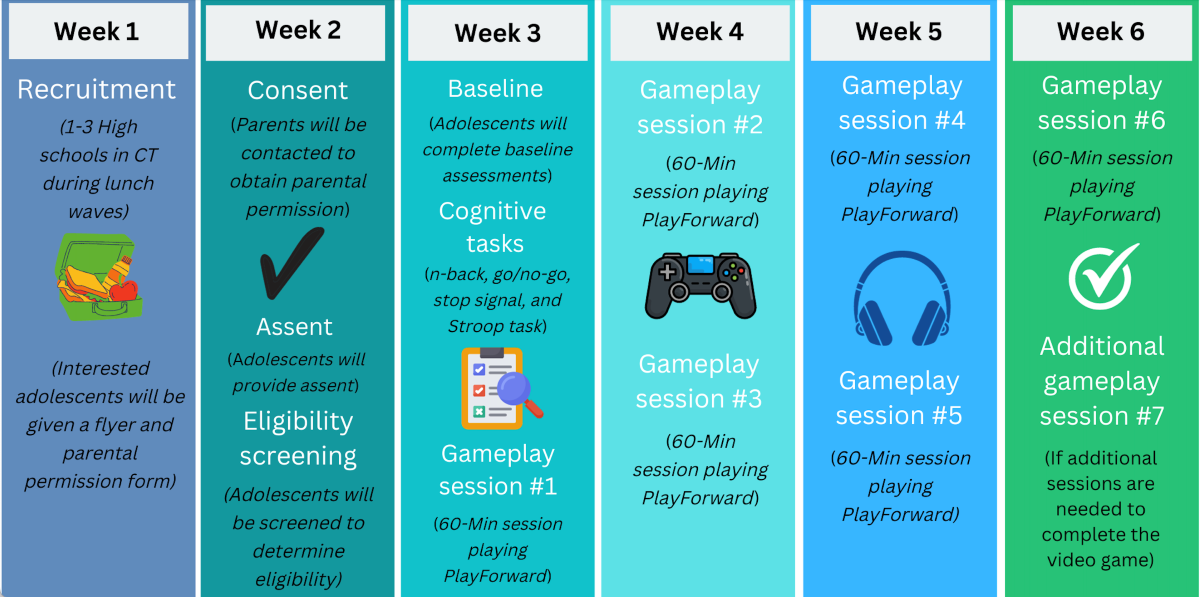
Aim 2 procedures. CT: Connecticut.

Figure 1 outlines study procedures. After obtaining verbal parental permission and adolescent assent, participants will be provided with their study ID to log-in to PsyToolkit to complete baseline procedures, which include 7 validated questionnaires and 4 performance-based cognitive tasks as described above.

Following the completion of baseline assessments or tasks, all enrolled participants will be provided a National Institutes of Mental Health–approved information sheet about the teen brain with resources on finding help. In addition, all participating schools have a school-based health center where students can seek help. Enrolled participants will use iPads to complete the entire *PlayForward* game over 6 to 7 sessions lasting 60 minutes each (the average number of 60-min sessions needed to complete the *PlayForward* game is 6). Gameplay sessions will occur 1 to 2 times/wk. The order in which the 4 cognitive tasks will be presented to participants will be random and generated by PsyToolkit. Adolescents will be compensated US $50 following the completion of baseline and US $70 after the completion of this study.

### Statistical Analyses

#### Primary Outcomes

Working memory: the percentage of correct matches, percentage of missed matches, and percentage of false alarms from the 2-back task will be used to compute a working memory score.

Inhibitory control: the response speed and number of errors from the go or no-go task and average response time from the stop-signal task will be used to compute an inhibitory control score.

#### Secondary Outcomes

Selective attention and processing speed: the average response time from the Stroop task will be used to compute the selective attention score.

#### Predictors

In-game performance metrics extracted in aim 1 will also be extracted in aim 2. We will test if specific in-game performance metrics are predictive of cognitive function (working memory and inhibitory control) measured by performance-based tasks in a sample of adolescents (n=30).

#### Planned Analyses

The model trained in aim 1 will be applied to the data collected in aim 2, and digital biomarker scores will be computed such that higher scores equate to higher risk for substance misuse. We will carry out a test of correlation (*r*) to determine the correlation between the digital biomarker score and (1) working memory score, (2) inhibitory control score, and (3) selective attention score.

#### Power

With an expected moderate correlation of 0.5 between in-game performance score and validated measures of cognitive function [28], a total of 29 participants will provide 80% power at the .05 2-sided significance level. A larger sample size will yield conventional levels of statistical significance for lower correlation coefficients (n=30 for *r*=0.5 and n=47 for *r*=0.4). For this pilot project, we anticipate a correlation of 0.5 based on prior validation studies comparing game-based cognitive assessment to standardized assessment [75-77].

### Ethical Considerations

The Yale institutional review board approved this study and its procedures on October 11, 2022 (IRB #2000033084). This study is covered by a Certificate of Confidentiality from the National Institutes of Health. Given the sensitive nature of this study, a Health Insurance Portability and Accountability Act waiver and waiver of written parental consent was obtained.

## Results

This study was funded by the National Institute on Drug Abuse through the Center for Technology and Behavioral Health at Dartmouth Pilot Core in May 2022. For aim 1, we completed cleaning of in-game log files in preparation for data extraction. In total, 166 log files were initially available for data extraction. Following data cleaning, 6 files were excluded for reasons such as the data storage files were corrupted, the data structure of log files was different from the rest of the files, and it was impossible to determine the reason for this difference. As such, a final sample of 160 is being used for analysis. The number of extracted in-game performance metrics is 285 (Multimedia Appendix 1). The R software [46] was used in extracting metrics from game log files, and codes are being stored on GitHub. Analyses are currently underway. For aim 2, data collection is ongoing and is projected to end in February 2024. Currently, we have enrolled 12 participants. Data analysis for aim 2 will begin once data collection is completed. The results from both aims will be reported in a subsequent publication, which we expect to publish in the winter of 2024.

## Discussion

Adolescent substance misuse is a predominant public health problem. However, most adolescents at risk for substance misuse are not identified early [78]. Early identification of high-risk adolescents can inform the delivery of targeted interventions that prevent the development of negative outcomes. However, identifying adolescents at risk for substance misuse is not routinely done due to a myriad of factors. Although mobile phones and wearable devices have been used to identify and measure digital biomarkers for health outcomes [24-27], the use of games is less widely studied. The use of game-based assessment for mental health purposes has been on the rise and may be used to capture potential biomarkers of health outcomes that can be used for screening and monitoring [22]. The use of games for risk identification offers a promising solution as these can be deployed without the need for provider supervision, can offer privacy to the adolescent completing the game-based screener, and has benefits over self-report data as it would provide a more objective assessment of risk.

In this proof-of-concept study, we will test the utility of in-game performance metrics—digital biomarkers—to identify adolescents at risk for substance misuse. We will also test the hypothesis that adolescents with poor cognitive function will perform worse in the game and be at higher risk for substance misuse. We will be assessing aspects of cognitive function that are implicated in the development of substance misuse and impacted by substance misuse. We build on prior studies that have successfully used game metrics to measure cognitive function [38,40] and extend prior knowledge in the field by applying it to substance misuse. Games offer an engaging way to assess a wide range of factors among adolescents, most of whom play games [17]. This study has several strengths. To the best of our knowledge, we will be the first to test the utility of in-game performance metrics to be used as digital biomarkers of substance misuse. We will also be the first to investigate game-based digital biomarkers in an adolescent population.

This study has several limitations. We are using a game that was not initially designed to measure cognitive function. As such, the game metrics may be poor representations of working memory and inhibitory control. However, other studies have demonstrated that performance metrics collected in a game could be useful in predicting cognitive functions [38,40]. We are using a game that is designed to change behavior related to substance use risk. As such, this game may not be ideal as an assessment tool since the tools’ predictive ability may wane with repeated use in the setting of positive behavior change. However, other studies have documented that even games designed primarily for assessment can change cognitive processes in ways that can threaten the validity of the game-based tool [79,80]. We chose to use this game-based intervention due to the availability of existing data for this proof-of-concept study, which, if successful, will provide preliminary data for future development of a game-based assessment tool for substance misuse risk. Another limitation is that the initial sample on which the prediction model will be trained has a low prevalence of substance misuse. This imbalance may limit the performance of the prediction models to predict substance misuse. To address this, we will use data augmentation methods such as Synthetic Minority Oversampling Technique [81]. In addition, our eligibility criteria for aim 2 will allow us to recruit a sample of adolescents with higher risk for substance use than in aim 1, which may enhance the performance of the model.

Despite limitations, this study represents a novel and likely highly impactful examination of the use of digital games as not only interventions but also potentially as screening and data collection tools. If found to be successful in predicting substance use, it will inform the future design of game-based assessment tools for substance misuse risk. With their broad level of reach and high engagement, games could seamlessly provide data on adolescent substance misuse risk and help to close the gap in screening and early intervention, leading to improved health outcomes in adolescents.

## Appendix

Multimedia Appendix 1 Extracted in-game performance metrics.

## Abbreviations

AUROC: area under the receiver operating characteristic
MCI: mild cognitive impairment

## Data Availability

The data set containing the relevant in-game metrics extracted are included in this published paper in Multimedia Appendix 1 (n=285).
